# Mental health and well-being of fathers of children with intellectual disabilities: systematic review and meta-analysis

**DOI:** 10.1192/bjo.2019.75

**Published:** 2019-11-07

**Authors:** Kirsty Dunn, Deborah Kinnear, Andrew Jahoda, Alex McConnachie

**Affiliations:** Research Assistant, Mental Health and Wellbeing Group, University of Glasgow, UK; Senior Lecturer, Mental Health and Wellbeing Group, University of Glasgow, UK; Professor, Mental Health and Wellbeing Group, University of Glasgow, UK; Professor, Mental Health and Wellbeing Group, University of Glasgow, UK

**Keywords:** Intellectual disability, carers, mental health

## Abstract

**Background:**

Caring for a child with intellectual disabilities can be a very rewarding but demanding experience. Research in this area has primarily focused on mothers, with relatively little attention given to the mental health of fathers.

**Aims:**

The purpose of this review was to summarise the evidence related to the mental health of fathers compared with mothers, and with fathers in the general population.

**Method:**

A meta-analysis was undertaken of all studies published by 1 July 2018 in Medline, PsycINFO, CINAHL and EMBASE, using terms on intellectual disabilities, mental health and father carers. Papers were selected based on pre-defined inclusion and exclusion criteria.

**Results:**

Of 5544 results, 20 studies met the inclusion criteria and 12 had appropriate data for meta-analysis. For comparisons of fathers with mothers, mothers were significantly more likely to have poor general mental health and well-being (standardised mean difference (SMD) −0.38, 95% CI −0.56 to −0.20), as well as higher levels of depression (SMD, −0.46; 95% CI −0.68 to −0.24), stress (SMD, −0.32; 95% CI −0.46 to −0.19) and anxiety (SMD, −0.30; 95% CI −0.50 to −0.10).

**Conclusions:**

There is a significant difference between the mental health of father and mother carers, with fathers less likely to exhibit poor mental health. However, this is based on a small number of studies. More data is needed to determine whether the general mental health and anxiety of father carers of a child with intellectual disabilities differs from fathers in the general population.

## Existing research on parent carers

There has been a growing body of research on parent carers' mental health and well-being.^1–3^ The majority of research in this area has focussed on maternal carers, with little known about the mental health of fathers of children with intellectual disabilities. Of those studies that included fathers in their sample, methodological limitations were evident, including small sample size,^1,4^ unclear information about sample (e.g. mental health not reported separately for mothers and fathers)^5–7^ and biased sampling (e.g. exclusion of fathers from analysis).^8,9^ The number of fathers taking on a caregiving role with their child has increased in recent decades^10^ and there is now a cultural expectation within the UK for father involvement.^11^ Given this shift in parental roles, it is vital to learn more about the impact of caring on fathers.

## ABCX model of stress

One model, which attempts to account for differences in father adaptation to stress, is the ABCX family crisis model.^12,13^ This model proposes that a stressor (A) is moderated by parental resources (B) and parental cognitions or perception of the stressor (C), to result in an outcome of stress or other indicator of adjustment (X). When this model is applied to the current area of research, the stressor refers to the birth of the child with intellectual disabilities. Policy makers and service providers cannot meet fathers' needs without reliable information on which factors affect their mental health. Therefore, a review of the literature is timely and will provide a comprehensive and up-to-date synthesis of the best evidence available on the mental health of fathers who care for a child with intellectual disabilities. A systematic review and meta-analysis was conducted to investigate the available research to date. The research questions were as follows:

- Does the mental health and well-being of father carers of a child with intellectual disabilities differ from fathers in the general population, or mother carers of a child with intellectual disabilities?
- Is the mental health and well-being of father carers of a child with intellectual disabilities moderated by paternal financial resources, paternal social support or parental perceptions of the characteristics of the child?

## Method

### Selection of studies

**Fig. 1.**
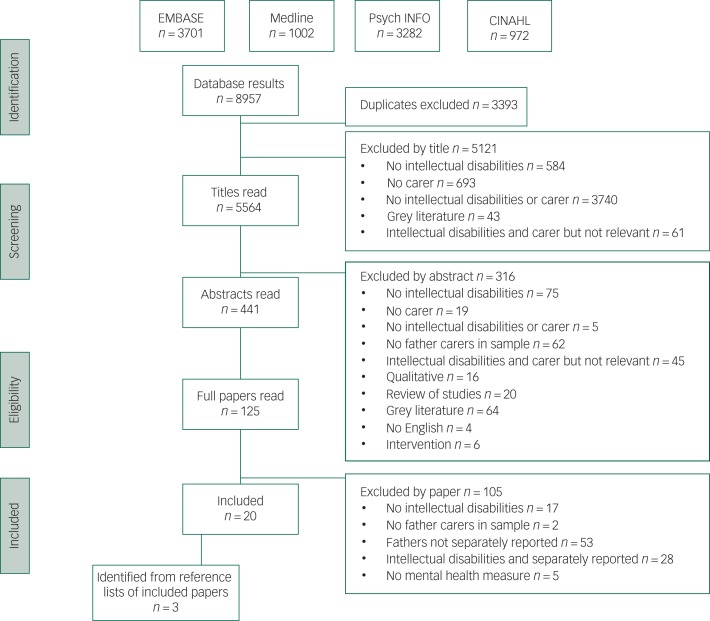
PRISMA flow diagram

The review was prospectively registered with the International Prospective Register of Systematic Reviews (PROSPERO, registration number: CRD42017075898). The Preferred Reporting Items for Systematic Reviews and Meta-Analyses (PRISMA) checklist was followed. The literature search was conducted on 1 July 2018. The specific search strategy included relevant terms for intellectual disabilities, carers and mental health (Supplementary File 1 available at https://doi.org/10.1192/bjo.2019.75). The following databases were searched: PsycINFO, EMBASE, Medline and CINAHL. The initial search was conducted by a single researcher, with a second researcher searching a random selection of the retrieved papers; 10% of titles and 10% of abstracts. The reasons for any discrepancies in paper selection were identified and resolved through discussion. Authors were contacted for further information where it was not clear if the study met the inclusion criteria. Reference lists and citations of included papers were also scrutinised. A PRISMA flow diagram^14^ was completed, detailing the reasons for excluding studies (Fig. 1).

A decision was taken by the authors to focus this systematic review and meta-analysis on papers with samples from countries of Western culture. This decision was influenced by a number of factors. Resources and services available to parents from countries in Eastern (e.g. India, Pakistan) and Western (e.g. Sweden, Australia) cultures are likely quite different. Countries with more traditional gender roles may also lead to fathers carrying out fewer caregiving activities than fathers in countries where cultural expectations lead to a more equal division of household tasks.^15^ This difference in involvement in caregiving could lead to different stress levels among fathers. Differing attitudes toward disability could also mean that more shame, for example, is attached to intellectual disabilities in particular cultures, leading to higher stress levels for parents.^16^ Following an examination of papers that met the initial inclusion criteria, sufficient papers were retrieved to justify separating the papers into those from Western and Eastern cultures. Papers which met the inclusion criteria and were from Eastern cultures will be reported in a separate systematic review and meta-analysis.

Strict inclusion and exclusion criteria were used to select papers.

#### Inclusion criteria

We included studies of fathers of children who had received a diagnosis of intellectual disability. Definitions of intellectual disability were accepted as an IQ ≥2 s.d. below the population mean. In studies where no IQ was provided, we accepted fathers of children who were described as having an ‘intellectual disability’, ‘learning disability’ or any of the equivalent terms set out on our list of search strategy (terms 1–5) in Supplementary File 1. Further inclusion criteria were fathers aged 16 years and above; fathers of all ethnicities; observational studies such as cohort, case–control and cross-sectional studies; accepted measures of fathers' mental health and well-being, including validated mental health and well-being measures (e.g. the Warwick–Edinburgh Mental Well-being Scale^17^) and validated measures of specific mental health conditions (e.g. Beck Depression Inventory^18^); studies from peer-reviewed journals; studies from Western countries and papers written in the English language.

#### Exclusion criteria

Exclusion criteria were studies where father carer's data were not separately reported, and studies where fathers of children with intellectual disabilities were among samples of fathers of children with other disabilities, but data of fathers of children with intellectual disabilities were not separately reported.

### Data extraction and quality assessment

Data from each study was extracted with a data extraction form to collect information about the author, publication year, country, setting, type of study, population characteristics, methodology, outcome measures, key findings and limitations of the study. When studies did not clearly meet the inclusion criteria, authors were contacted to request additional information or clarification.

We assessed the quality of all the selected studies in a systematic way, ensuring we covered all the domains included in a systematic review of tools to assess quality of observational studies.^19^ This included the clarity of the stated aims, methods (including age/gender standardisation and whether group differences in disease prevalence rates were considered), design, participant selection, study size, measures used, data collected, analyses used, results, biases, generalisability, conflicts of interests and ethical procedures. Additionally, to generate a ‘score’, we added up the number of items on the Oxford Critical Appraisal Skills Programme (CASP) Checklist^20^ that were addressed in each study. For example, ‘the cohort was recruited in an acceptable way’. For each CASP item the authors indicated whether the study under evaluation had high, low or uncertain risk in this category. If the cohort was recruited in a suitable manner then this item was rated as low risk; if it was not or the recruitment methods were not clearly stated in the paper then the study was rated as being of high or uncertain risk. The CASP scores assigned to each included study are displayed in Supplementary Tables 1–4. The reliability of the appraisal was checked by two of the authors.

The following classification was given to rate the risk of bias for each study overall: rating A, low risk of bias for all 14 items; rating B*x*, uncertain risk of bias for *x* items and low risk of bias in all other items; and rating C*y,x*, high risk of bias in *y* items, uncertain risk of bias in *x* items and low risk of bias in all other items (e.g. a paper with high risk of bias on two items, uncertain risk of bias on three items and low risk of bias on all other items would receive a rating of C2,3).

Initially, descriptive analysis of the studies was completed. Meta-analysis was undertaken using Review Manager 5.3 (RevMan 5.3) software for Mac (The Nordic Cochrane Centre, The Cochrane Collaboration, Copenhagen, Denmark, 2014; see https://community.cochrane.org/help/tools-and-software/revman-5/revman-5-download/installation). All outcome measures from the included studies reported outcomes on a continuous scale. The mean, s.d. and number of individuals in the sample of father carers and the comparison group sample were extracted from each paper, and the unbiased standardised mean difference (SMD) was calculated. A negative mean difference indicated poorer mental health for the comparison group than for father carers. For papers that included more than one group of father carers (e.g. fathers of children with Down syndrome and fathers of children with fragile-X syndrome), data for these two groups were entered separately into the meta-analysis because data for these groups was reported separately, and this would allow for comparisons between groups. Effect size was interpreted as follows: SMD < 0.40 indicated small effect size, SMD 0.40 to 0.70 indicated moderate effect size and SMD > 0.70 indicated large effect size.

The chi-squared statistic *I*^221^ was used to indicate how much heterogeneity was present across the studies. It is not influenced by how many studies are in the meta-analysis, unlike some other test statistics, and can be interpreted in a similar manner regardless of the type of outcome data or effect measurement. It is therefore appropriate for this analysis as the included papers used a range of mental health measures. Higgins and colleagues propose that 0% equals no heterogeneity, 25% equals low heterogeneity, 50% equals medium heterogeneity and 75% equals high heterogeneity. Random effects models were selected for this analysis because of the different populations (e.g. fathers of children with different types of intellectual disabilities, fathers of different ages) and measures (e.g. different measures of mental health) used in the included studies. Funnel plots were used to assess the effect of publication bias.

### Sensitivity analysis

The impact of studies' risk of bias rating on the pooled SMD was ascertained by sensitivity analysis. This was done by removing data from the meta-analysis for each included study one by one, and beginning with the lowest ranked papers, to determine the effect of each individual study on the pooled SMD.

### Subgroup analysis

The analysis was done by different subgroups based on different mental health conditions, including anxiety, depression, stress and general mental health and well-being. A further subgroup analysis was conducted after removing papers where fathers made up <50% of the sample.

## Results

A total of 22 papers were initially retrieved with the search strategy that met the inclusion criteria. The flow chart documents the number of papers included/excluded at each stage after reading titles, abstracts and full papers, and the reasons for exclusions (Fig. 1). The first and second researchers fully agreed on all the titles and abstracts to be included at these stages, so further discussions were not required. Three authors were contacted and responded to requests for additional information.^22–25^ As a result of acquiring further information from the authors, the papers from Glidden and colleagues^22,23^ were excluded from the review, bringing the total number of included studies to 20.

Of the 20 studies, 12 met the inclusion criteria,^24–36^ and had appropriate data for meta-analysis. The remaining eight papers met the systematic review inclusion criteria but their results were not suitable for meta-analysis and are presented descriptively. Each of the research questions are addressed below with a descriptive analysis, followed by the results of the meta-analysis, where this was possible. As the papers used different measures of mental health, the included papers which address possible differences in mental health between father carers and other populations were divided into subgroups. These subgroups were based on type of mental health condition reported in the paper: depression, stress, anxiety and general mental health. A further subgroup analysis was then conducted after removing papers where <50% of the sample were fathers. Risk rating scores for each study included in the meta-analysis are detailed in Supplementary Table 1.

### The mental health and well-being of father carers of a child with intellectual disabilities compared with fathers in the general population

Regarding the first research question, Supplementary Table 2 presents the nine studies which report the impact of caring on fathers of children with intellectual disabilities compared with fathers in the general population.^27,29,31,35,37–41^ The outcome measures used in these studies were the Centre for Epidemiologic Studies Depression Scale,^42^ the Swedish version of the SF-36,^43^ the Beck Depression Inventory,^18^ the Distress Thermometer for Parents,^44^ the Hospital Anxiety and Depression Scale^45^ and the Parental Perception Inventory.^46^ These studies were from Australia, the UK, Ireland, the Netherlands and Sweden.

Olsson and Hwang^38^ compared fathers of children under 16 years of age with and without intellectual disabilities. Although no difference in depression scores was found between groups, the five remaining included studies did report a significant difference in mental health scores. Hedov *et al*^37^ measured self-reported mental health in fathers of children with and without Down syndrome aged 3.5 to 7 years of age. Fathers of children with Down syndrome reported significantly worse mental health than fathers of typically developing children. A later study by Hedov *et al*^40^ compared responses from fathers of young children (aged 3–7 years) with Down syndrome and fathers of typically developing children on the Parental Perceptions Inventory. On three of the inventory's 20 items, fathers of children with Down syndrome experienced significantly higher stress levels. In Olsson and Hwang's^31^ study, depression scores of fathers of children under 17 years of age with intellectual disabilities were compared with a general population comparison group in Sweden. Results of this comparison found that father carers' depression scores were higher than fathers whose child did not have intellectual disabilities. Emerson *et al*^39^ compared fathers of children with severe and less severe cognitive delay to fathers of children with no delay when their child was aged 3 and 5 years. Fathers of children with severe delay were more likely to have poor mental health than fathers of children with no delay only when their child was 5 years old. Fathers whose child had less severe delay were more likely to be at risk of poor mental health than fathers of children with no delay when their child was 3 and 5 years old. In MacDonald *et al*’s^41^ study, fathers of children with Down syndrome (mean age 11 years) reported higher levels of depression and anxiety than fathers whose child did not have an intellectual disability. Norlin and Broberg^29^ compared the mental health of fathers with young children who did or did not have intellectual disabilities. Depression and stress scores were higher for fathers whose child had intellectual disabilities. In Giallo *et al*'s^27^ study, fathers of children with intellectual disabilities (aged 3–15 years) reported significantly higher levels of depression and stress, but not anxiety, than normative data for the Australian adult general population. Marchal *et al*^35^ compared outcomes on the Distress Thermometer for Parents in fathers of children (aged 11–13 years) with Down syndrome to fathers in the general population. Clinical distress was reported more frequently by fathers of children with Down syndrome than by the control group.

Data necessary for conducting a meta-analysis was not available in Hedov *et al*'s^37^ study, Hedov *et al*'s^40^ study, Olsson and Hwang's^38^ study, Macdonald *et al*'s^41^ study, Marchal *et al*'s^35^ study and Emerson *et al*'s^39^ study. Hedov *et al*^37^ measured general mental health whereas Olsson and Hwang^31^ measured depression, and Norlin and Broberg^29^ and Giallo *et al*^27^ measured depression and stress. Therefore, it was only possible to conduct a meta-analysis of depression and stress scores reported by father carers compared with the general population. The control group in Giallo *et al*'s^27^ study was the general population of Australian adults, including both fathers and mothers. The mental health of fathers was not reported separately. Therefore, a meta-analysis of depression scores was conducted both with and without this study to compare the impact of this study on the meta-analysis results. The pooled SMD for depression between father carers and fathers in the general population (when including Giallo *et al*'s^27^ study) was −0.24 (95% CI −0.35 to −0.13, *P* < 0.001). There was evidence of statistical heterogeneity between the studies for the meta-analyses (*I*^2^ = 0%). The pooled SMD for depression between father carers and fathers in the general population (when excluding Giallo *et al*'s^27^ study) was −0.22 (95% CI −0.39 to −0.04, *P* < 0.001). There was no evidence of statistical heterogeneity between the studies for the meta-analyses (*I*^2^ = 0%), and the effect size was small. The pooled SMD for stress between father carers and fathers in the general population was 0.61 (95% CI 0.05–1.17, *P* < 0.05). There was a moderate effect size and evidence of a high level of heterogeneity, with *I*^2^ = 89%.

### The mental health and well-being of father carers compared with mother carers of children with intellectual disabilities

The 17 studies presented in Supplementary Table 3 compared the mental health and well-being of mothers and fathers with a child with intellectual disabilities.^25–38,40,47,48^ The studies took place in Sweden, Australia, Poland, the UK, the USA and the Netherlands. The outcome measures used in these studies were the Depression Anxiety and Stress Scale,^49^ General Health Questionnaire,^50^ Hassles and Uplifts Scale,^51^ Parenting Daily Hassles Scale,^52^ Centre for Epidemiologic Studies Depression Scale,^42^ Questionnaire on Resources and Stress,^53^ Short Form Health Survery-36,^43^ Beck Depression Inventory,^18^ Perceived Stress Scale,^54^ Distress Thermometer for Parents,^44^ Brief Symptom Inventory^55^ and Parental Perception Inventory.^46^

Fourteen of these studies reported poorer mental health and well-being for mothers compared with fathers of people with intellectual disabilities. In Giallo *et al*'s^27^ Australian study, mothers reported significantly higher depressive, anxiety and stress scores than fathers. Mothers in Gerstein *et al*’s^26^ study of children with intellectual disabilities reported significantly higher scores on the Parenting Daily Hassles Scale than fathers when their child was aged 48 months and 60 months. In Giallo *et al*'s^27^ study, mothers reported significantly higher depressive, anxiety and stress scores than fathers. Depression, but not stress scores, were significantly higher for mothers than fathers in Norlin and Broberg's^29^ study. Olsson and Hwang^31^ also reported that mother well-being was more affected than father well-being when there was a child with intellectual disabilities in the family. A number of other studies in Sweden by Olsson and Hwang also found poorer mental health in mothers than fathers.^31,32,38^ Mothers of children aged 5 years and under with intellectual disabilities had lower levels of well-being than fathers.^32^ Mother well-being was also more affected than father well-being when there was a young child with intellectual disabilities in the family.^31^ A third study by the authors found that mothers of children under 16 years of age had higher depression scores than fathers.^38^ Another Swedish study by Hedov *et al*^37^ reported that fathers of children aged 3.5 to 7 years of age with Down syndrome had poorer mental health scores than mothers.^37^

In Stoneman's^34^ study mothers reported higher rates or depression and stress than fathers. Griffith *et al*’s^28^ study of children aged 2–19 years with a range of intellectual disabilities also found poorer mental health for mothers than fathers, although the extent of this difference varied by type of intellectual disability and well-being measure. Dabrowska and Pisula^47^ also reported higher stress levels for mothers than fathers of children with Down syndrome. Marchal *et al*^35^ compared total problem scores on the Distress Thermometer for Parents of mothers and fathers of children with Down syndrome. Mothers reported significantly more problems than fathers.

A different pattern was reported by a number of other included papers. No significant difference was found between stress or general mental health scores for mothers and fathers of children with fragile-X syndrome (Mccarthy *et al*^36^). The mean score for mothers and fathers on the psychological distress scale was not in the clinical range and so these parents may be coping better than other parents of children with fragile-X syndrome. Kózka *et al*^48^ found no significant difference in psychological well-being between mothers and fathers of children with Down syndrome. Foster *et al*^25^ found that mothers reported significantly poorer overall well-being than fathers of children with Smith–Magenis syndrome. Parents reported similar levels of anxiety but fathers reported significantly higher levels of depression than mothers. However, this sample contained 97 mothers and only 15 fathers as a comparison group, and so definitive conclusions cannot be drawn as these fathers may not be representative of other fathers within this population. Rowbotham *et al*’s^33^ study found no significant difference in mental health scores between mothers and fathers of children with intellectual disabilities. Both groups reported a very high level of symptoms, with the majority of parents falling within the clinical range. As the sample size of this study was very small, it is possible that this paper differs from the other included studies as the selected group was not representative of other parents of children with intellectual disabilities in Australia. The children included in this study may also not be representative of those who exhibit challenging behaviour, a factor associated with parental well-being, as their adaptive and problem behaviour scores generally fell within the normal range. This may indicate that the children had milder intellectual disabilities than children in the other samples included in this review, although level of intellectual disabilities was not reported in this paper.

#### Meta-analysis

All studies with the necessary data provided in the paper were included in the meta-analysis^25–37^ of the general mental health of father and mother carers of children with intellectual disabilities, which included measures of general mental health and well-being, depression, anxiety and stress.

As the studies focussed on different mental health conditions (depression, anxiety, stress, general mental health and well-being), separate analyses were conducted to compare studies which studies reported each of these conditions individually. The pooled SMD indicated that mothers were significantly more likely to have general mental health problems than fathers (−0.26 95% CI −0.47 to −0.06, *P* < 0.01); this was also the case for depression (−0.47 95% CI −0.68 to −0.25, *P* < 0.001), stress (−0.29, 95% CI −0.42 to −0.15, *P* < 0.001) and anxiety (−0.30, 95% CI −0.51 to −0.10, *P* < 0.001) The effect size was moderate for depression and small for general mental health, stress and anxiety. There was no evidence of statistical heterogeneity between studies in the general mental health, anxiety or stress meta-analyses, with *I*^2^ = 0% in each analysis. For the depression meta-analysis, *I*^2^ = 64%, and for the general mental health meta-analysis, *I*^2^ = 34%, and so there is a moderate level of heterogeneity between studies.

A subanalysis was conducted on studies where fathers made up 50% or more of the sample. The pooled SMD for general mental health between father and mother carers was −0.38 (95% CI −0.63 to −0.13, *P* < 0.01). The pooled SMD for depression was −0.49 (95% CI −0.64 to −0.34, *P* < 0.001), the SMD for stress was −0.31 (95% CI −0.48 to −0.15, *P* < 0.001) and the SMD for anxiety was −0.36 (95% CI −0.62 to −0.11, *P* < 0.01). There was no evidence of statistical heterogeneity between studies for any of the meta-analyses, with *I*^2^ = 0% in each analysis. The effect size was moderate for depression and small for general mental health, stress and anxiety.

See Supplementary File 2 for the meta-analysis forest and funnel plots. There was no evidence of publication bias for any of the mental health conditions included in the meta-analyses.

#### Risk of bias

Supplementary Table 1 provides the risk of bias details for each of the 12 papers included in the meta-analysis. There were three studies with a B1 rating,^32,37,41^ four studies with a B2 rating,^26,30,31,36^ two with a B3 rating,^27,28^ one with a C1,2 rating^25,33^ and one with a C2,2 rating.^35^ In all papers, the factors that returned a rating of ‘unclear risk’ were deemed to be of limited concern, as there were only an average 2.0 out of 14 items that were unclear in any one paper and all authors agreed that the unclear items did not represent high risk. All papers were therefore considered reliable evidence.

#### Sensitivity analysis

Sensitivity analysis in relation to risk of bias was run for the 12 studies included in the meta-analysis (Supplementary File 3). Studies were removed in order of risk-of-bias rating, and studies with smaller samples were removed first where multiple studies had the same rating. The pooled SMD for depression scores changed slightly as the lowest rated studies (rated C1,3 to C1,2) were removed from the analysis (from −0.47 to −0.56). For the studies which reported stress scores, pooled SMD did not change with the removal of the included C1,3 study. The pooled SMD for anxiety scores changed slightly as the lowest rated studies were removed from the analysis (from −0.30 to −0.37). For the studies which reported general mental health scores, pooled SMD changed with the removal of the lowest rated studies (from −0.26 to −0.22).

### Factors that moderate the mental health and well-being of father carers of a child with intellectual disabilities

Supplementary Table 4 displays the studies which address the second research question as to which factors moderate the impact of caring on fathers.

#### Paternal financial resources

Seven studies investigated whether paternal financial resources moderate the mental health and well-being of father carers of a child with intellectual disabilities.^27,29,31,32,34,39,41^ These studies took place in Poland, the USA, Ireland, Australia and Sweden. Degree of participation in paid employment was used as a measure of financial resources in Olsson and Hwang's^31^ study, which found that fathers' well-being increased with higher involvement in paid employment. Giallo *et al*^27^ also reported that not being in paid employment was significantly associated with increased anxiety among fathers. However, this did not significantly predict the variance in stress, or depression in fathers. These mixed results may be attributable to broad single-item measures, which may not have fully captured family economic status.

Parental financial resources or socioeconomic status were measured in the other four studies. Stoneman^34^ also found that reports of depression by fathers of children with intellectual disabilities were predicted by lower family income. In Olsson and Hwang's^32^ study, socioeconomic hardship was one of the strongest predictors of paternal mental ill-health. Emerson *et al*^39^ found that matching fathers with and without a child with cognitive delay on the basis of socioeconomic circumstances reduced between-group differences in the prevalence of fathers' psychiatric disorders from 45%, to 11%. In fact, differences between fathers of children with different types of intellectual disabilities disappeared after income differences between the groups were controlled. Norlin and Broberg^29^ found that reports of poor mental health were associated with high levels of economic hardship in fathers. The age of parents in these studies ranged from early 30's to early 70's. Macdonald *et al*^41^ reported that fathers whose partner worked outside the family home displayed lower rates of anxiety than those whose partner was not employed. It was not possible to conduct a meta-analysis with the above studies as sufficient data was only available in the Olsson and Hwang paper.^32^

#### Paternal social support

Supplementary Table 4 also reports the four studies that addressed the effect of social support provided by a partner or spouse.^26,29,48,56^ These studies were from Sweden, Poland and the USA. Gerstein *et al*^26^ reported that father reported marital adjustment when their child was 36 months old served as a protective factor for fathers' mental health. Marital quality also predicted well-being for fathers in Norlin and Broberg's^29^ study of parents in Sweden. Stress was significantly related to reported marital quality for fathers of children with Down syndrome (Norton *et al*^56^). However, Kózka *et al*^48^ found no significant relationship between marital quality and psychological well-being of fathers of children with Down syndrome. In contrast to the other included studies, Kózka *et al*^48^ used a combined score of mother and father rated marital quality rather than the fathers' marital satisfaction score alone, which may account for this difference. Again, it was not possible to conduct a meta-analysis with the above studies because of a lack of comparable data.

#### Type of intellectual disabilities

Four studies compared fathers of different types of intellectual disabilities.^24,28,34,41^ These studies were from the USA, the UK and Ireland. In Hartley *et al*’s^24^ study, a significant difference was found between fathers of adolescents with Down syndrome, fragile-X syndrome and autism spectrum disorder. Fathers of children with autism spectrum disorder reported significantly higher levels of depressive symptoms than those whose child had Down syndrome or fragile-X syndrome. No significant difference was found between fathers of children with Down syndrome and fragile-X syndrome. Stoneman^34^ reported lower levels of depression for fathers whose children had Down syndrome than those whose children had another type of intellectual disability, such as Prader–Willi syndrome and fragile-X syndrome. In the study by Griffith *et al*^28^, the well-being of fathers whose children were aged 2–19 years and had Angelman, Cornelia de Lange, or *cri du chat* syndromes were compared. Poorest mental health was reported for fathers of children with Angelman syndrome, followed by *cri du chat* syndrome and then Cornelia de Lange syndrome. MacDonald *et al*^41^ also found fathers of children aged 6–19 years with Down syndrome reported lower levels of stress than fathers of children with other types of intellectual disability.

#### Level of intellectual disabilities

One study compared the impact of level of intellectual disability on fathers' mental health and well-being.^39^ These studies were conducted in the UK. Emerson *et al*^39^ compared parental mental health when their child was 3 and then 5 years of age. Fathers of a child with severe cognitive delay were more likely to be at risk of poor mental health than fathers of a child with no delay only when the child was 5 years old. Fathers of a child with less severe cognitive delay were more likely to be at risk of poor mental health than fathers of a child with no delay when the child was both 3 and 5 years old.

#### Challenging behaviour

Challenging behaviour was taken into account when assessing paternal mental health in six studies.^24,27,29,34,36,41^ These papers were from Australia, Sweden and the USA. The study by Stoneman^34^ reported that having a child with a more difficult temperament predicted father depression scores. Challenging behaviour also significantly contributed to well-being in fathers of young children with intellectual disabilities in Norlin and Broberg's^29^ study. In Giallo *et al*'s^27^ study, child behaviour difficulties were identified as significantly predicting father stress and depressive and anxiety symptoms. Fathers of children with fragile-X syndrome experienced more stress when their child was exhibiting challenging behaviour (McCarthy *et al*^36^). This factor was the strongest predictor of stress for fathers in this study. Child behaviour problems were also strongly associated with father adjustment measures in Macdonald *et al*’s^41^ study.

However, behaviour problems were not significantly associated with paternal depressive symptoms in Hartley *et al*’s^24^ study. In Hartley *et al*’s^24^ study, mothers rather than fathers reported the challenging behaviour, which may account for this difference between studies. By contrast, fathers independently reported challenging behaviour in the other three papers. Because of the lack of information on challenging behaviour in the included studies, it is not possible to take this factor into consideration in this meta-analysis.

#### Coping strategies

Four studies investigated the effect of coping strategies that fathers use.^24,41,47,48^ The two coping strategies examined in Hartley *et al*’s^24^ study were emotion-focused and problem-focused coping. Emotion-focused coping was defined as ‘efforts to manage emotions surrounding the problem, e.g. trying to wish away negative feelings’ and problem-focused coping was defined as ‘efforts to alter the stressor itself, e.g. seeking information’. The well-being of fathers of children with Down syndrome, fragile-X syndrome and autism were compared. Although there was a significant difference in mental health between the groups, there was no significant difference in the use of coping strategy by diagnostic group of paternal psychological well-being. Dabrowska and Pisula^47^ also compared coping strategies in fathers whose children had Down syndrome, autism or were typically developing. There was no significant difference in coping styles between groups.

MacDonald *et al*^41^ reported that psychological acceptance partially mediated the impact of challenging behaviour on father stress, anxiety and depression. However, the study did not compare acceptance to other coping methods. Kózka *et al*^48^ measured the ego resiliency of fathers of children with Down syndrome, which they define as an inner mental structure that helps people to cope with difficult situations. Fathers who demonstrated higher levels of ego resiliency reported better psychological well-being than fathers who displayed lower levels of this coping style.

#### Other factors identified in the included studies

One study addressed the impact that the child has on the father, and its relationship to the father's mental health. Giallo *et al*^27^ reported that fathers' own needs, stress arising from child behaviour and needs, and low parenting satisfaction significantly predicted depressive, anxiety and stress symptoms. In Foster *et al*’s^25^ study, parents were asked to indicate their level of agreement with statements about the benefits of caring for a child with intellectual disabilities. Fathers who reported more benefits were more likely to have higher levels of carer well-being. Maternal depressive symptoms were associated with fathers' well-being in a study by Hartley *et al*^24^. Higher levels of maternal depressive symptoms were a significant positive predictor of paternal depression. Norton *et al*^56^ found a significant relationship between receiving regular respite and father daily stress. Fathers whose child with Down syndrome was regularly looked after by grandparents, babysitters, etc, reported lower stress levels than fathers who did not receive such respite.

## Discussion

### Mental health of mothers and fathers

The majority of studies that compared the mental health of father and mother carers^26–32,34,35,37,38,47^ indicated that there is a difference between the mental health of father and mother carers, with fathers exposed to a lower risk of depression, anxiety, stress and poor general mental health. Despite apparent changes in father's roles within society, the results of this review indicate that they continue to play a limited role in care. There are a number of possible reasons for the existence of this gender difference in mental health. Mothers more often work part time or give up work entirely to become the main caregiver for their child, whereas fathers who remain in the family unit are more often the main breadwinner.^57^ Given this difference, it is important to consider whether barriers exist within the workplace that could play a role in discouraging fathers from participating in care. A recent survey of over 8000 UK residents reported that workplace attitudes and policies reduced their ability to be as involved in caring as their partner. For example, 35% of employed fathers of children under 18 years of age said that men in their workplace who take time off to care for children are not supported.^58^ However, studies have shown that even in situations where the father is unemployed and the mother is employed, fathers often still function as the secondary caregiver and participate in fewer caregiving activities than mothers.^59–61^ This raises important questions about fathers' ability or desire to take part in caregiving activities, as well as how this is shaped by public attitudes and governmental or employment policies.

Time spent outside the family home in a different role may also contribute to gender differences in parental mental health. The results of Olsson and Hwang's^31^ study indicate a positive relationship between involvement in paid work and well-being for both mothers and fathers. Macdonald *et al*^41^ also reported that partner working outside the family home was associated with lower rates of anxiety in fathers. These results are also supported by later studies that found participation in the workforce to have a protective effect on mothers of children with intellectual disabilities.^62,63^ If mothers are less often involved in paid work following the birth of a child with intellectual disabilities, this could in part explain the difference in mental health between parents. In Olsson and Hwang's^31^ study, there was no difference in well-being between mothers who worked full time and mothers who worked part time. This suggests that taking a longer break from child care activities does not affect mother mental health, but that being in paid employment to some degree does have a protective effect. Their study also found that participation in paid employment made a significant contribution to the variance in well-being between fathers and mothers, but division of child care tasks did not. Participation in paid work can serve as a protection against social isolation and life dissatisfaction,^59^ and as fathers are more likely to work, this may contribute to their well-being.

### Paternal resources

The results of papers included in this review showed support for some factors as mediators of mental health and well-being for father carers. There was support for the impact of paternal financial resources on father carer mental health.^27,29,31,32,34,39,41^ It is not surprising that financial resources are linked to poor mental health as families on a lower income will be more limited in what they can provide for their children than families on a higher income. These findings replicate work on mothers of children with intellectual disabilities. In a study of mothers of children with severe intellectual disabilities, socioeconomic position moderated associations between child problem behaviours and maternal anxiety and depression.^64^ Social support, as measured by marital adjustment, was another factor that was associated with better mental health in all included studies.^26,29,48,56^ This finding is also supported by previous research on mothers of a child with intellectual disabilities, such as a large study which found that social support and parental stress had a significant negative correlation.^65^

### Child characteristics

The effect of the characteristics of their child was also explored. Four papers reported on the effect of type of intellectual disability.^24,28,34,41^ It has been documented that parents of children with Down syndrome have tended to score higher on well-being measures than parents of children with other types of intellectual disabilities.^66–68^ This pattern has been termed the Down syndrome advantage.^69^ The paper by Stoneman^34^ also reported lower levels of depression for fathers who had a child with Down syndrome, than those who had a child with another type of intellectual disabilities.^34^ Various explanations have been proposed to account for this pattern, such as parents of children with Down syndrome in previous samples have been older and of a higher income bracket than parents in the comparison groups.^69,70^ There is a well-established link between low income and mental health problems in the general population.^72^ The Stoneman^34^ study supports this explanation as the Down syndrome advantage disappeared after socioeconomic factors were controlled. Hartley *et al*^24^ also found lower mental health scores for fathers of adolescents with fragile-X syndrome than fathers of adolescents with Down syndrome. However, this became non-significant once father age, child behaviour problems, mother mental health and number of children with disabilities in the family were controlled for. This finding is also supported by existing research, which documents that children with Down syndrome usually display lower levels of challenging behaviour than other types of intellectual disabilities, and that this is linked to parental well-being.^70,71^

The impact of level of intellectual disability on father mental health was also reported. In Emerson *et al*'s^39^ study, British fathers of children with severe cognitive delay had poorer mental health than fathers whose child had no delay, whereas there was no difference between fathers whose child had less severe cognitive delay and had no delay. Young children with more severe intellectual disabilities have more significant support needs, and there is more of a cultural expectation for British fathers to be involved in providing support for their child and family.

The association between challenging behaviour and fathers' mental health was only reported in six studies, five of which found a positive relationship,^27,29,34,36,41^ and one of which found no such association.^24^ The negative impact of challenging behaviour on parental mental health is well documented in the literature,^71–76^ and studies that investigate the mental health of family carers but do not take account of challenging behaviour fail to capture an important variable.

### Strengths and limitations

This study has a number of strengths. To the best of our knowledge, this is the first meta-analysis on the mental health and well-being of father carers of a child with intellectual disabilities, and the factors that affect fathers' mental health. A robust method was employed and the quality of the included papers is high (rated B1 to C2,2 on risk of bias).

However, there were a limited number of studies that met the inclusion criteria, which restricted the number and type of meta-analyses that could be run. There were also various difficulties with directly comparing the results of the studies in this review. For example, the studies that addressed this research question used a variety of outcome measures, making direct comparisons between the papers difficult, as measures may vary in how they define mental ill-health.

It was also not possible to generate accurate age categories as many of the included papers did not report the age of their sample, and those that did focussed predominantly on middle-aged parents. Of the studies that did report a mean age for parents and children in their sample, the mean age of the father ranged from 37.0 to 43.0 years, and the mean age of the child with intellectual disabilities ranged from 4.8 to 10.5 years of age. There is some evidence that carers experience different levels of poor mental health throughout the caregiving journey, such as after receiving a diagnosis or at times of increased child care responsibility.^77,78^ As a result, the findings of this meta-analysis may only apply to middle-aged fathers with a young child, rather than younger or older father carers.

The majority of the studies included in this systematic review and meta-analysis recruited parents exclusively through early intervention programmes or services provided for families of children with disabilities. As a result, the majority of parents in the studies were known to services and so the results of our study may only be applicable to parents who exhibit help-seeking behaviour or have a greater level of need than other parents of a child with intellectual disabilities. A further limitation is that the vast majority of fathers in the included papers were those who had remained in the family unit. As marital/partner breakdown is common within families of children with intellectual disabilities, there is a disproportionate number of absent fathers compared with the general population. The results of this review may therefore not be representative of the mental health and well-being of absent fathers of children with intellectual disabilities.

### Future research

Studies that investigate risk and protective factors for father mental health have reported that the factors they investigated were more strongly associated with the mental health of mothers than fathers. That risk factors for such studies are generally drawn from previous research partly explains this pattern, as such research has largely focussed on mothers. More research on fathers is needed to identify factors that are important for predicting paternal well-being. This review also demonstrated the tendency for previous research to focus on risk, rather than protective factors. Further research is required to learn more about factors that serve protective functions for fathers.

Despite changing gender roles and societal expectations, the results of this review indicate that fathers of children and young adults with intellectual disabilities continue to play a small role in caregiving. This finding raises important questions about whether fathers want to be involved in caring, and if so, then what barriers they experience. Such information could assist policy makers and service providers to improve services and supports for fathers. Therefore, it is important for future research to explore the mental health and well-being of father carers of people with intellectual disabilities.

## Supplementary material

For supplementary material accompanying this paper visit http://dx.doi.org/10.1192/bjo.2019.75. click here to view supplementary material
