# Supplementary material for: Mental health and well-being of fathers of children with intellectual disabilities: systematic review and meta-analysis
Source: BJPsych Open. 2019 Nov 7;5(6):e96. doi: 10.1192/bjo.2019.75 (PMC6854361; doi:10.1192/bjo.2019.75)
Supplement: Supplementary file 1 [file S2056472419000759sup001.docx]

Table 1 Detailed CASP Scores for studies included in the meta-analysis

| Study | CASP Item | Level of Risk | Details |
| --- | --- | --- | --- |
| Foster *et al* (2010) **Score = C1, 2** | Was the cohort recruited in an acceptable way? | Low | Recruited through parent and researcher Smith-Magenis syndrome list serve. |
|  | Was the exposure accurately measured to minimise bias? | Unclear | ID measure was not reported. |
|  | Was the outcome accurately measured to minimise bias? | Low | The Center for epidemiologic studies depression scale, Becks Anxiety Inventory and Caregiver well-being scale were used, which are commonly used self-report measures. |
|  | Have the authors identified all important confounding factors? | High | Level of child’s ID was not reported, which could be a confounding factor. The sample size of fathers was also very small. |
|  | Have the authors taken account of the confounding factors in the design? | Low | Recruitment method may have resulted in only families who seek help being identified. |
|  | Was the follow up of subjects complete enough? | Low | N/A |
|  | Was the follow up of subjects long enough? | Low | N/A |
|  | Did the study address a clearly focused issue? | Low | The study question was focussed. It was clear which population was studied and which outcome measures were selected. |
|  | What are the results of the study? | Low | Results are adequately reported. |
|  | How precise are the results? | Unclear | Confidence intervals not given. |
|  | Do you believe the results? | Low | Results are supported by prior studies. |
|  | Can the results be applied to the local population? | Low | No evidence to suggest that the participants were sufficiently different from the population to cause concern. |
|  | Do the results of this study fit with other available evidence? | Low | The results are supported by prior studies. |
|  | What are the implications of this study for practice? | Low | Implications for practice are considered by the authors. |
| Gerstein (2009) **Score = B2** | Was the cohort recruited in an acceptable way? | Low | The families were from a longitudinal study. The sample was recruited from community agencies serving families with children with ID, so it may not represent families not accessing these services but the risk is small. |
|  | Was the exposure accurately measured to minimise bias? | Low | Children in the study were assessed for ID through their Mental Development Index score and BSID-II measure. Both are commonly used to measure mental development in children. These were administered at home. |
|  | Was the outcome accurately measured to minimise bias? | Low | Daily Parenting Hassle measure was used to measure stress, which is commonly used self-report measure. |
|  | Have the authors identified all important confounding factors? | Unclear | Parental age and level of child’s ID were not reported, which could be confounding factors. |
|  | Have the authors taken account of the confounding factors in the design? | Low | Recruitment method may have resulted in only families who seek help being identified. |
|  | Was the follow up of subjects complete enough? | Low | N/A |
|  | Was the follow up of subjects long enough? | Low | N/A |
|  | Did the study address a clearly focused issue? | Low | The study question was focussed. It was clear which population was studied and which outcome measures were selected. |
|  | What are the results of the study? | Low | Results are adequately reported. |
|  | How precise are the results? | Unclear | Confidence intervals not given. |
|  | Do you believe the results? | Low | Results are supported by prior studies. |
|  | Can the results be applied to the local population? | Low | No evidence to suggest that the participants were sufficiently different from the population to cause concern. |
|  | Do the results of this study fit with other available evidence? | Low | The results are supported by prior studies. |
|  | What are the implications of this study for practice? | Low | Implications for practice are considered by the authors. |
| Giallo (2015) **Score = B3** | Was the cohort recruited in an acceptable way? | Unclear | The sample was recruited from families involved in a previous study of children in a challenging behaviour programme. These families were identified through schools and advertisement in newsletters of organisations specializing in support for families with children with ID. Sample contained some children who had both ID and ASD. |
|  | Was the exposure accurately measured to minimise bias? | Low | The measure of ID was not reported in this study although all children had been assessed as having ID. |
|  | Was the outcome accurately measured to minimise bias? | Low | The Depression Anxiety and Stress Scale was used, which is a well-known measure. Based on mental health in the past week. |
|  | Have the authors identified all important confounding factors? | Unclear | Children all had challenging behaviour, so results may be different for children without challenging behaviour. Also demonstrates that families have sought help. These were identified by the authors. |
|  | Have the authors taken account of the confounding factors in the design? | Unclear | Acknowledged but design not altered to account for this. |
|  | Was the follow up of subjects complete enough? | Low | N/A |
|  | Was the follow up of subjects long enough? | Low | N/A |
|  | Did the study address a clearly focused issue? | Low | The study question was focussed. It was clear which population was studied and which outcome measures were selected. |
|  | What are the results of the study? | Low | Results are adequately reported. |
|  | How precise are the results? | Low | Confidence intervals are not wide. |
|  | Do you believe the results? | Low | Results are supported by previous studies. |
|  | Can the results be applied to the local population? | Low | The results can be applied to other families whose child has ID and challenging behaviour. |
|  | Do the results of this study fit with other available evidence? | Low | Results are supported by previous studies. |
|  | What are the implications of this study for practice? | Low | Implications for practice and future research are considered. |
| Griffith (2011) **Score = B3** | Was the cohort recruited in an acceptable way? | Low | The families were recruited from a pre-existing database held by the research team and through national parent syndrome support groups. |
|  | Was the exposure accurately measured to minimise bias? | Low | Parents reported that their child had been diagnosed with ID. |
|  | Was the outcome accurately measured to minimise bias? | Low | Questionnaire on Resources and Stress short form, Hospital Anxiety and Depression Scales, and Genetic Syndrome Stressors Scale, which are commonly used self-report measures. |
|  | Have the authors identified all important confounding factors? | Unclear | Level of child’s ID were not reported, which could be a confounding factor. |
|  | Have the authors taken account of the confounding factors in the design? | Low | Recruitment method may have resulted in only families who seek help being identified. Small sample sizes for each of the syndromes, although this is partly because the syndromes are rare and so difficult to recruit these parents. |
|  | Was the follow up of subjects complete enough? | Low | N/A |
|  | Was the follow up of subjects long enough? | Low | N/A |
|  | Did the study address a clearly focused issue? | Low | The study question was focussed. It was clear which population was studied and which outcome measures were selected. |
|  | What are the results of the study? | Low | Results are adequately reported. |
|  | How precise are the results? | Unclear | Confidence intervals not given. |
|  | Do you believe the results? | Low | Results are supported by prior studies. |
|  | Can the results be applied to the local population? | Unclear | Due to the small number in the sample it is difficult to say if the sample is sufficiently different from the population to cause concern. |
|  | Do the results of this study fit with other available evidence? | Low | The results are supported by prior studies. |
|  | What are the implications of this study for practice? | Low | Implications for practice are considered by the authors. |
| Hedov (2000) **Score = B1** | Was the cohort recruited in an acceptable way? | Low | Randomly recruited through child’s paediatrician. |
|  | Was the exposure accurately measured to minimise bias? | Low | Parents reported that their child had been diagnosed with Down Syndrome. |
|  | Was the outcome accurately measured to minimise bias? | Low | Swedish version of the SF36, which is a commonly used self-report measure. |
|  | Have the authors identified all important confounding factors? | Low | Level of child’s ID were not reported, which could be a confounding factor. |
|  | Have the authors taken account of the confounding factors in the design? | Low | No. |
|  | Was the follow up of subjects complete enough? | Low | N/A |
|  | Was the follow up of subjects long enough? | Low | N/A |
|  | Did the study address a clearly focused issue? | Low | The study question was focussed. It was clear which population was studied and which outcome measures were selected. |
|  | What are the results of the study? | Low | Results are adequately reported. |
|  | How precise are the results? | Unclear | Confidence intervals not given. |
|  | Do you believe the results? | Low | Results are supported by prior studies. |
|  | Can the results be applied to the local population? | Low | There is no reason to believe the sample is sufficiently different from the population to cause concern. |
|  | Do the results of this study fit with other available evidence? | Low | The results are supported by prior studies. |
|  | What are the implications of this study for practice? | Low | Implications for practice are considered by the authors. |
| MacDonald (2010) **Score = B1** | Was the cohort recruited in an acceptable way? | Low | Randomly recruited through ID services. |
|  | Was the exposure accurately measured to minimise bias? | Low | Receipt of service provision from recruitment organisations was conditional on having an ID. |
|  | Was the outcome accurately measured to minimise bias? | Low | Questionnaire on resources and stress, which is a commonly used self-report measure. |
|  | Have the authors identified all important confounding factors? | Low | Level of child’s ID and parent age were not reported, which could be confounding factors. |
|  | Have the authors taken account of the confounding factors in the design? | Low | No |
|  | Was the follow up of subjects complete enough? | Low | N/A |
|  | Was the follow up of subjects long enough? | Low | N/A |
|  | Did the study address a clearly focused issue? | Low | The study question was focussed. It was clear which population was studied and which outcome measures were selected. |
|  | What are the results of the study? | Low | Results are adequately reported. |
|  | How precise are the results? | Unclear | Confidence intervals not given. |
|  | Do you believe the results? | Low | Results are supported by prior studies. |
|  | Can the results be applied to the local population? | Low | There is no reason to believe the sample is sufficiently different from the population to cause concern. |
|  | Do the results of this study fit with other available evidence? | Low | The results are supported by prior studies. |
|  | What are the implications of this study for practice? | Low | Implications for practice are considered by the authors. |
| Marchal *et al* (2017) **Score = C2,2** | Was the cohort recruited in an acceptable way? | Low | Recruited from participation in a medication trial for people with DS. |
|  | Was the exposure accurately measured to minimise bias? | Unclear | ID measure was not reported. |
|  | Was the outcome accurately measured to minimise bias? | Low | The Distress Thermometer for Parents was used, which is a commonly used self-report measure. |
|  | Have the authors identified all important confounding factors? | High | Level of child’s ID was not reported and only parents of ‘healthy’ children were included, which could be confounding factors. |
|  | Have the authors taken account of the confounding factors in the design? | Low | No |
|  | Was the follow up of subjects complete enough? | Low | N/A |
|  | Was the follow up of subjects long enough? | Low | N/A |
|  | Did the study address a clearly focused issue? | Low | The study question was focussed. It was clear which population was studied and which outcome measures were selected. |
|  | What are the results of the study? | Low | Results are adequately reported. |
|  | How precise are the results? | Unclear | Confidence intervals not given. |
|  | Do you believe the results? | Low | Results are supported by prior studies. |
|  | Can the results be applied to the local population? | High | Parents of children with an Apgar score below normal were excluded and so this is a ‘healthy’ subgroup of parents of children with DS. |
|  | Do the results of this study fit with other available evidence? | Low | The results are supported by prior studies. |
|  | What are the implications of this study for practice? | Low | Implications for practice are considered by the authors. |
| McCarthy (2010) **Score = B2** | Was the cohort recruited in an acceptable way? | Low | Randomly recruited through Fragile X society. |
|  | Was the exposure accurately measured to minimise bias? | Unclear | Fragile X was reported by parents. |
|  | Was the outcome accurately measured to minimise bias? | Low | The Brief Symptom Inventory was used, which is a commonly used self-report measure. |
|  | Have the authors identified all important confounding factors? | Low | Level of child’s ID was not reported and parents were asked to report on their child with the highest support needs, which could be confounding factors. |
|  | Have the authors taken account of the confounding factors in the design? | Low | Confounding factors are considered. |
|  | Was the follow up of subjects complete enough? | Low | N/A |
|  | Was the follow up of subjects long enough? | Low | N/A |
|  | Did the study address a clearly focused issue? | Low | The study question was focussed. It was clear which population was studied and which outcome measures were selected. |
|  | What are the results of the study? | Low | Results are adequately reported. |
|  | How precise are the results? | Unclear | Confidence intervals not given. |
|  | Do you believe the results? | Low | Results are supported by prior studies. |
|  | Can the results be applied to the local population? | Low | There is no reason to believe the sample is sufficiently different from the population to cause concern. |
|  | Do the results of this study fit with other available evidence? | Low | The results are supported by prior studies. |
|  | What are the implications of this study for practice? | Low | Implications for practice are considered by the authors. |
| Olsson (2002) **Score = B2** | Was the cohort recruited in an acceptable way? | Low | The sample was recruited from community based programmes providing services to families of disabled children. |
|  | Was the exposure accurately measured to minimise bias? | Unclear | Children in the study were assessed for ID through parent reports. |
|  | Was the outcome accurately measured to minimise bias? | Low | Beck’s Depression Inventory was used, which is a commonly used self-report measure. |
|  | Have the authors identified all important confounding factors? | Low | Level of child’s ID was not reported, which could be a confounding factor. Takes other factors into account. |
|  | Have the authors taken account of the confounding factors in the design? | Low | Recruitment method may have resulted in only families who seek help being identified. |
|  | Was the follow up of subjects complete enough? | Low | N/A |
|  | Was the follow up of subjects long enough? | Low | N/A |
|  | Did the study address a clearly focused issue? | Low | The study question was focussed. It was clear which population was studied and which outcome measures were selected. |
|  | What are the results of the study? | Low | Results are adequately reported. |
|  | How precise are the results? | Unclear | Confidence intervals not given. |
|  | Do you believe the results? | Low | Results are supported by prior studies. |
|  | Can the results be applied to the local population? | Low | No evidence to suggest that the participants were sufficiently different from the population to cause concern. |
|  | Do the results of this study fit with other available evidence? | Low | The results are supported by prior studies. |
|  | What are the implications of this study for practice? | Low | Implications for practice are considered by the authors. |
| Olsson (2006) **Score = B2** | Was the cohort recruited in an acceptable way? | Low | The sample was recruited from community based programmes providing services to families of disabled children. |
|  | Was the exposure accurately measured to minimise bias? | Unclear | Children in the study were assessed for ID through parent reports. |
|  | Was the outcome accurately measured to minimise bias? | Low | Beck’s depression inventory was used, which is a commonly used self-report measure. |
|  | Have the authors identified all important confounding factors? | Low | Level of child’s ID was not reported, which could be a confounding factor. Takes other factors into account. |
|  | Have the authors taken account of the confounding factors in the design? | Low | Recruitment method may have resulted in only families who seek help being identified. |
|  | Was the follow up of subjects complete enough? | Low | N/A |
|  | Was the follow up of subjects long enough? | Low | N/A |
|  | Did the study address a clearly focused issue? | Low | The study question was focussed. It was clear which population was studied and which outcome measures were selected. |
|  | What are the results of the study? | Low | Results are adequately reported. |
|  | How precise are the results? | Unclear | Confidence intervals not given. |
|  | Do you believe the results? | Low | Results are supported by prior studies. |
|  | Can the results be applied to the local population? | Low | No evidence to suggest that the participants were sufficiently different from the population to cause concern. |
|  | Do the results of this study fit with other available evidence? | Low | The results are supported by prior studies. |
|  | What are the implications of this study for practice? | Low | Implications for practice are considered by the authors. |
| Olsson (2008) **Score = B1** | Was the cohort recruited in an acceptable way? | Low | The sample was recruited from community based programmes providing services to families of disabled children. |
|  | Was the exposure accurately measured to minimise bias? | Low | Parents reported that their child had been diagnosed with intellectual disabilities. |
|  | Was the outcome accurately measured to minimise bias? | Low | Version of the Beck’s Depression Inventory (BDI-2r) used to measure mental health, which is commonly used self-report measure. |
|  | Have the authors identified all important confounding factors? | Low | Level of child’s ID were not reported, which could be a confounding factor. |
|  | Have the authors taken account of the confounding factors in the design? | Low | Recruitment method may have resulted in only families who seek help being identified. |
|  | Was the follow up of subjects complete enough? | Low | N/A |
|  | Was the follow up of subjects long enough? | Low | N/A |
|  | Did the study address a clearly focused issue? | Low | The study question was focussed. It was clear which population was studied and which outcome measures were selected. |
|  | What are the results of the study? | Low | Results are adequately reported. |
|  | How precise are the results? | Unclear | Confidence intervals not given. |
|  | Do you believe the results? | Low | Results are supported by prior studies. |
|  | Can the results be applied to the local population? | Low | There is no evidence that the sample is sufficiently different from the population to cause concern. |
|  | Do the results of this study fit with other available evidence? | Low | The results are supported by prior studies. |
|  | What are the implications of this study for practice? | Low | Implications for practice are considered by the authors. |
| Rowbotham (2011) **Score = C1,3** | Was the cohort recruited in an acceptable way? | Low | The sample was recruited from public and private-sector groups providing services to carers of adults with ID. |
|  | Was the exposure accurately measured to minimise bias? | Unclear | Children in the study were assessed for ID through the Adaptive Behavioural Scale which was carried out by parents. |
|  | Was the outcome accurately measured to minimise bias? | Low | The General Health Questionnaire-28, which is commonly used self-report measure. |
|  | Have the authors identified all important confounding factors? | High | Very small sample size. |
|  | Have the authors taken account of the confounding factors in the design? | Low | Recruitment method may have resulted in only families who seek help being identified. |
|  | Was the follow up of subjects complete enough? | Low | N/A |
|  | Was the follow up of subjects long enough? | Low | N/A |
|  | Did the study address a clearly focused issue? | Low | The study question was focussed. It was clear which population was studied and which outcome measures were selected. |
|  | What are the results of the study? | Low | Results are adequately reported. |
|  | How precise are the results? | Unclear | Confidence intervals not given. |
|  | Do you believe the results? | Low | Results are supported by prior studies. |
|  | Can the results be applied to the local population? | Unclear | No evidence to suggest that the participants were sufficiently different from the population to cause concern, but the sample size was very small. |
|  | Do the results of this study fit with other available evidence? | Low | The results are supported by prior studies. |
|  | What are the implications of this study for practice? | Low | Implications for practice are considered by the authors. |

Table 2

| Author & Year | Father Characteristics | Comparison Group | Son/daughter with/without ID | Study Design | Measures | Methodology | Results | Critique |
| --- | --- | --- | --- | --- | --- | --- | --- | --- |
| Marchal *et al* (2017) | *N* = 44 DS fathers Mean age 47.8 (s.d. 5.4) The Netherlands | *N* = 52 TD fathers | *N* = 44 DS, 52 TD Age 11–13 yrs | Cross sectional | ID: -Parent report Parental Mental Health: -The Distress Thermometer for Parents | Recruited from participation in a medication trial for people with DS | Total distress score was significantly higher for DS fathers M 6.3 (s.d. 5.9) *v.* TD fathers M 3.5 (s.d. 4.8) | CASP Score = C2,2 -ID measure not reported -Level of ID not reported -Only parents of ‘healthy’ children with DS were included, so may not be representative -95%CI not reported |
| Giallo *et al* (2015) | *N* = 315 fathers Age not reported Australia | Men and women in general population *N* = 497 Age not reported | 315 children with ID plus one other condition (ASD, vision/hearing impairment, DS, cerebral palsy, epilepsy, psychiatric disorder, ADHD, brain injury, health problem) Mean age 7.8 yrs (s.d. 5.5) 69.8% male ID: 17.1% mild, 7.3% moderate, 2.2% severe, 0.3% profound, 73.1% not reported | Cross sectional | ID: -measure not reported Parental Mental Health: -Depression Anxiety and Stress Scale -Developmental Behaviour Checklist | Recruited from families involved in a previous study (Hudson *et al*, 2003; 2008). In the original study families recruited through schools and advertisement in newsletters of organizations specializing in support for families with children with ID | -Fathers in the sample reported sig higher depression and stress than mothers and fathers in the general population. Depression: M 6.95, s.d. 7.89 *v.* M5.02, s.d. 7.54 t = 4.35, cohens d = 0.25 (CI 0.11–0.39). Anxiety: M 3.49, s.d. 5.24 *v.* M 3.36, s.d. 5.07 t = 0.42, cohens d = 0.03 (CI −0.12–0.17). Stress: M 11.0, s.d. 8.24 *v.* M 8.10 s.d. 8.40 t = 6.24, cohens d = 0.35 (CI 0.21–0.49) | CASP Score = B3 -Father sample from those enrolled to participate in a child behaviour management programme- so not be representative -Includes children with ID + ASD in the sample -Mental health score based on distress in the past week |
| Norlin *et al* (2013) | *N* = 46 ID fathers Mean age 35.3 (s.d. 6.1) Sweden | *N* = 141 TD fathers Mean age 36.4 (s.d. 5.8) | *N* = 58 ID Mean age 41 months (s.d. 27.8) 62.1% males *N* = 182 TD Mean age 41.3 months (s.d. 20.5) 55.8% males | Cross- sectional | *ID:* *-Not reported* *Parental Mental Health:* *-BDI-2r* | ID: Recruited by staff at service centres for families of children with disabilities TD: Nationwide register | Depression scores were higher for ID than TD fathers: −21.5 (s.d. 11.2) *v.* −23.3 (s.d. 12.5) Stress scores were higher for ID than TD fathers: 9.9 (s.d. 8.1) *v.* 5.1 (s.d. 3.8) | CASP Score = B2 -Did not report ID measure used -Confidence intervals not reported |
| Emmerson *et al* (2010) | Wave one: *N* = 18 552 families Wave two: *N* = 15 590 families Wave three: *N* = 15 246 families Age not reported UK |  | Wave one =  9 mths old Wave two =  3 yrs old Wave three = 5 yrs old | Longitudinal | ID: -Bracken Basic Concept Scale -Naming Vocabulary subscale from British Ability Scale II Parental Mental Health: -K6 Scale | Secondary data analysis of Millennium Cohort Study (waves 1–3) Families eligible to receive child benefit, born 09/2000 and 01/2002 | Severe Delay: Fathers more likely to be at risk of psychiatric disorder than fathers of no delay only when child was 5 years old (14% *v.* 8%, OR = 1.82, 95%CI 1.05–3.15, *P* < 0.031) Less severe delay: Fathers more likely to be at risk of psychiatric disorder than fathers of no delay only when child was 3 (14% *v.* 7%, OR = 1.93, 95% CI = 1.10–3.42, *P* = 0.021) and 5 years old (15% *v.* 8%, OR = 1.99, 95%CI 1.10–3.61, *P* = 0.020). | CASP Score = B3 - Young children in sample so possible not all those with ID have been identified -Follow-up not long enough to identify ID diagnosed later in development -Level of ID not reported -Parent age not reported |
| MacDonald *et al* (2010) | *N* = 53 DS fathers Age not reported Ireland | *N* = Males from large, community, non-clinical sample of males (Crawford *et al*. 2001) | *N* = 99 children Mean age 11 yrs 10 months (s.d. 42.85) | Cross sectional | ID: -Not reported Parental Mental Health: -Positive Gain Scale -Parent and Family Problems subscale of Questionnaire on Resources and Stress -Hospital Anxiety and Depression scale | Recruited from ID services | Fathers *v.* General Population Males Depression: 7.1% *v.* 2% Anxiety: 10.1% *v.* 8% | CASP Score = B1 -95%CI’s not reported -Level of ID not reported -parent age not reported |
| Olsson *et al* (2006) | *N* = 375 fathers 179 ID fathers mean age 43 yrs (s.d. 6.3) Sweden | 196 TD fathers mean age 42 yrs (s.d. 6.9) | *N* = 179 ID mean age 8.1 yrs (s.d. 4.3) 62% males *N* = 196 TD mean age 8.2 yrs (s.d. 4.4) 62% males | Cross-sectional | ID: -Parent reports Parental Mental Health: -Beck’s Depression Inventory -Division of 15 child-care tasks measure | Recruited from community-based programmes providing services to families of disabled children | ID carer wellbeing was lower than TD fathers: 4.1 (s.d. 4.8) *v.* 5.4 (s.d. 5.7) | CASP Score = B2 -ID measured with parent reports -Level of ID not reported |
| Hedov *et al* (2002) | *N* = 79 DS fathers Mean age 39.6 (s.d. 5.9) Sweden | *N* = 82 TD fathers Mean age 38.3 (s.d. 5.8) | *N* = 79 DS 82 TD Age DS 3.5–7 yrs (M 4.7) *N* = 87 TD children Age 4–6 (M 4.7) | Cross sectional | ID: -Not reported Parental Mental Health: -Parental Perception Inventory | Recruitment method not reported | 3 of 20 stress items were sig different between DS and TD fathers: ‘Extra demands on my time’ was higher for DS fathers *P* < 0.01 ‘Feel stress when thinking of child’s future’ was higher in DS fathers, *P* < 0.0005 ‘Finding someone to stay with child’ was higher in DS fathers, *P* < 0.025 | CASP Score = B3 -Recruitment method not reported -ID measure not reported -95%CI not reported |
| Olsson *et al* (2001) | *N* = 135 120 ID fathers Age not reported Sweden | 185 TD fathers | *N* = 151 ID Mean age 7.4 yrs (s.d. 4.2) 60% males *N* = 496 TD Mean age 8.4 yrs (s.d. 2.2) 63% males | Cross- sectional | ID: -Medical provider’s classification Parental Mental Health: -Becks Depression Inventory | Recruited from community-based programmes for families of children with disabilities | ID Fathers reported higher BDI scores than TD fathers: 5 *v.* 4.1 This was not significant | CASP Score = B2 -Recruited from community programmes so may not represent families not accessing services -Parents age not reported -Confidence intervals not reported |
| Hedov *et al* (2000) | *N* = 179 *N* = 79 DS fathers Mean age 39.6 (s.d. 6.0) Sweden | *N* = 100 TD fathers Mean age 36 (s.d. 5.6) | *N* = not reported DS Mean age 4.7 yrs *N* = not reported Under 18 yrs | Cross- sectional | ID: -Not reported Parental Mental Health: -SF 36 mental health domain | Recruited through paediatrician | DS fathers were significantly more affected in the mental health domain of the SF36: 79.2 (s.d. 17.9) *v.* 87.1 (s.d. 16) *P* < 0.002 | CASP Score = B1 -Confidence intervals not reported |

Table 3

| Author & Year | Father Characteristics | Comparison Group | Son/daughter with/without ID | Study Design | Measures | Methodology | Results | Critique |
| --- | --- | --- | --- | --- | --- | --- | --- | --- |
| Koza *et al* (2018) | *N* = 51 fathers Mean age 48.31 Poland | *N* = 75 mothers Mean age 45.33 | *N* = DS Mean age 13.25 48.41% Male | Cross sectional | ID: -Not reported Parental Mental Health: -Perceived Stress Scale -Oxford Happiness Questionnaire | Recruited from schools, rehabilitation centres and previous contacts | No significant difference in psychological well-being between mothers and fathers *P* = 0.094 | CASP Score = B3 -ID measure not reported -ID level not reported -95%CI not reported |
| Marchal *et al* (2017) | *N* = 44 fathers Mean age 47.8 (s.d. 5.4) The Netherlands | *N* = 76 mothers Mean age 45.9 (s.d. 4.1) | *N* = 86 DS 11–13 yrs | Cross sectional | ID: -Not reported Parental Mental Health -The Distress Thermometer for Parents | Recruited from participation in a medication trial for people with DS | Mothers *v.* Fathers Total problem score on Distress Thermometer: M 7.5 (s.d. 6.7) *v.* M 6.3 (s.d. 5.9) | CASP Score = C2,2 -A healthy subgroup of children with DS were used -ID measure not reported -Leve of ID not reported -95%CI not reported |
| Giallo *et al* (2015) | *N* = 315 fathers Age not reported Australia | 1) Mothers from same family (subset of *N* = 110) Age not reported | 315 children with ID plus one other condition (ASD, vision/hearing impairment, DS, cerebral palsy, epilepsy, psychiatric disorder, ADHD, brain injury, health problem) Mean age 7.8 yrs (s.d. 5.5) 69.8% male ID: 17.1% mild, 7.3% moderate, 2.2% severe, 0.3% profound, 73.1% not reported | Cross sectional | ID: -Not reported Parental Mental Health: Depression Anxiety and Stress Scale -Developmental Behaviour Checklist -Parenting Hassles Scale -Parenting Sense of Competence Scale | Recruited from families involved in a previous study (Hudson *et al*, 2003; 2008). In the original study families recruited through schools and advertisement in newsletters of organizations specializing in support for families with children with ID | Mother and father in same family *N* = 220: Depression: M 6.95 (s.d. 7.89) *v.* M 5.02 (s.d. 7.54) t = 4.35, Cohens d = 0.25 (CI 0.11–0.39). Anxiety: M 3.49, s.d. 5.24 *v.* M 3.36, s.d. 5.07 t = 0.42, Cohens d = 0.03 (CI −0.12–0.17). Stress: M 11.0, s.d. 8.24 *v.* M 8.10 s.d. 8.40 t = 6.24, Cohens d = 0.35 (CI 0.21–0.49) | CASP Score = B3 -Father sample from those enrolled to participate in a child behaviour management programme- so not representative -Includes children with ID + ASD in the sample -Mental health score based on distress in the past week |
| Norlin *et al* (2013) | *N* = 46 fathers Mean age 35.3 (s.d. 6.1) Sweden | *N* = 58 mothers Mean age 34.0 (s.d. 5.3) | *N* = 58 Mean age 41 months (s.d. 27.8) 62.1% males | Cross sectional | ID: -Not reported Parental Mental Health: -BDI-2r | Recruited by staff at service centres for families of children with disabilities | Depression scores were significantly higher for mothers than fathers: −12.4 (s.d. 3.8) *v.* −21.5 (s.d. 11.2), *P* = 0.01 Stress scores were not significantly different for mothers and fathers: 10.8 (s.d. 8.2) *v.* 9.9 (s.d. 8.1) | CASP Score = B2 -Did not report ID measure used -Confidence intervals not reported |
| Griffiths *et al* (2011) | *N* = 39 Fathers: -Angelman syndrome = 12 Mean age 42.38 (s.d. 4.82) -Cornelia de Lange syndrome = 14 Mean age 47.6 (s.d. 10.38) -Cri du chat syndrome = 13 Mean age 41.92 (s.d. 4.92) UK | *N* = 47 mothers -Angelman syndrome = 14 Mean age 41.79 (s.d. 6.04) -Cornelia de Lange syndrome = 15 Mean age 47.31 (s.d. 8.90) -Cri du chat syndrome = 18 Mean age 39.56 (s.d. 5.22) | Angelman syndrome *N* = 15 Mean age 10.07 (4.79) Cornelia de Lange syndrome *N* = 16 Mean age 11.75 (s.d. 3.49) Cri du chat syndrome *N* = 18 Mean age 7.83 (s.d. 4.66) | Cross- sectional | ID: -Vineland adaptive behaviour scale (VABS-II) Parental Mental Health: -Questionnaire on Resources and Stress-Short form | Recruited from a database held by research team, and national parent support groups | M(mother) F(father) Anxiety: -Angelman syndrome M 11.71 (s.d. 3.97) *v.* F 10.42 (s.d. 4.72) -Cornelia de Lange M 8.93 (4.73) *v.* F 5.85 (s.d. 4.35) -Cri du Chat M 9.49 (2.90) *v.* F 9.0 (s.d. 4.16) Depression -Angleman syndrome M 8.57 (s.d. 3.08) *v.* F 8.50 (s.d. 4.49) -Cornelia de Lange M 7.30 (5.03) *v.* F 4.29 (s.d. 2.62) -Cri du Chat M 7.36 (3.42) *v.* F 6.92 (s.d. 4.09) Stress -Angleman syndrome M 26.31 (s.d. 8.17) *v.* F 20.75 (s.d. 10.07) -Cornelia de Lange M 19.76 (8.78) *v.* F 16.18 (s.d. 98.67) -Cri du Chat M 20.94 (7.23) *v.* F 16.68 (s.d. 5.52) | CASP Score = B3 -Level of ID not reported -Confidence intervals not reported -Small number of fathers in sample |
| Rowbotham *et al* (2011) | *N* = 12 fathers 45–55 yrs (with the exception of one father aged 55–65 yrs) Australia | *N* = 12 mothers from same family 45–55 yrs | *N* = 12 children Mean age 24 yrs (range: 18–30 yrs) 58% females Problem behaviour exhibited by the children did not differ between mothers and fathers, and was within the average range. | Cross- sectional | ID: -Adaptive Behavioural Scale (as reported by parents) Parental Mental Health: -Carers’ Assessment of Difficulties Index (CADI) - Carers’ Assessment of Satisfactions Index (CASI) - Hassles and Uplifts Scale - General Health Questionnaire-28 (GHQ-28) | Recruited through public and private-sector groups providing services to carers of adults with intellectual disabilities | -CADI & CASI Both mothers and fathers reported experiencing significantly more satisfaction than difficulties in their care-giving (mothers: Z = 2.76, *P* = 0.006; fathers: Z = 2.22, *P* = 0.026) and mothers reported more Uplifts than Hassles (mothers: Z = 2.18, *P* = 0.029) but the difference for fathers was not significant (fathers: Z = 1.56, *P* = 0.119). -GHQ-28 Total Score in clinical range for: 11/12 fathers (Mean 16.33 (s.d. 5.74)) and 11/12 mothers (Mean 16.58 (s.d. 8.07)) Wilcozon Z −0.05 = No significant differences. GHQ-28 Subscales: No significant differences Severe depression: Fathers mean 1.33 (s.d. 2.10), mothers mean 1.33 (s.d. 0.89) Wilcoxon Z −1.46. Social dysfunction: Fathers mean 5.25 (s.d. 2.18), mothers mean 6.17 (s.d. 1.64) Wilcoxon Z-1.47 Anxiety/Insomnia: Fathers mean 4.83 (s.d. 3.07) mothers mean 4.00 (s.d. 2.97) Wilcoxon Z −0.77 Somatic: Fathers mean 5.71 (s.d. 3.61) mothers mean 4.17 (s.d. 2.44) Wilcozon Z 0.00 No significant differences between mothers and fathers on any of the sub-scales of the GHQ-28. - Adult child problem behaviours were unrelated with GHQ-28 total scores for both mothers and fathers. | CASP Score = B4 -Small sample of parents known to services -Parents carried out ID measure -Level of ID not reported -Confidence intervals not reported |
| Dabrowska *et al* (2010) | DS *N* = 27 Mean age 34.9 (s.d. 6.1) Poland | DS *N* = 27 Mean age 32.8 (s.d. 6.1) | N not reported −55.6% males Mean age 4.3 (s.d. 1.58) −44.4% females Mean age 4.3 (s.d. 1.48) | Cross- sectional | Parental Mental Health: -Questionnaire of Resources and Stress (QRS) - Coping Inventory for Stressful Situations (CISS) Socioeconomic Factor: -Questionnaire of Resources and Stress for Families with Chronically Ill or Handicapped Members short form | Recruited from centres for early intervention (DS), therapy centres (autism) or kindergar- tens (DS and TD). | Mothers of children with Down Syndrome experienced higher stress levels than fathers | CASP Score = B3 -95%CI not reported -Small sample size -Level of ID not reported -Young children in sample so possible not all those with ID have been identified. |
| Foster *et al* (2010) | *N* = 15 fathers Mean age 42.07 yrs (s.d. 9.85) USA | *N* = 97 mothers Mean age 41.36 yrs (s.d. 9.6) | *N* = Not reported | Cross sectional | ID: Not reported Parental Mental Health: -Center for epidemiologic studies depression scale -Becks Anxiety Inventory -Caregiver well-being scale | Recruited through parent and researcher Smith-Magenis syndrome list serve | Fathers and mothers both reported moderate/severe levels of depression (scores 24+): 43.7 (s.d. 12.2) *v.* 36.7 (s.d. 11.6) Fathers and mothers both reported moderate levels of anxiety (scores 22–35): 31.8 (s.d. 9.2) *v.* 30.9 (s.d. 8.5) Fathers reported lower levels of caregiver wellbeing than mothers: 135.8 (s.d. 21.5) *v.* 154.0 (s.d. 27.0) | CASP Score = C1,2 -Very small number of fathers in sample -Confidence intervals not reported -Level of ID not reported |
| Gerstein *et al* (2009) | *N* = 115 fathers Age not reported (married couples) USA | *N* = 115 mothers Age not reported | *N* = 115 ID 3 yrs old | Longitudinal (child aged 36–60 months) | ID: -Bayley Scales of Infant Development II Parent Mental Health: -Parenting Daily Hassles measure -Symptom Checklist-35 -Dyadic Adjustment Scale -Parent Child Interaction Rating Scale (tested at 36, 48, 60 months) | Recruited from community agencies serving families of children with ID | Mothers reported sig higher Parenting Daily Hassles score than fathers at 48 [t(80) = 3.366, *P* = 0.001] and 60 months [t(72) = 3.462, *P* = 0.001] | CASP Score = B2 -Level of ID not reported -Parental age not reported -95%CI not reported |
| Olsson *et al* (2008) | *N* = 49 fathers Mean age 36.2 yrs (s.d. 6.4) Sweden | *N* = 62 mothers Mean age 34.8 yrs (s.d. 6.0) | *N* = 62 0–5 yrs | Cross- sectional | ID: -Reported by centres Parental Mental Health: -Beck’s Depression Inventor-2r | Recruited from centres providing support to families of children with disabilities | Fathers had significantly lower BDI scores than mothers: −21.4 (s.d. 10.9) *v.* −12.4 (s.d. 13.9) Interaction effect of gender and ID (F1,429 = 5.7, *P* = 0.01) Mothers: High well-being 67.7% Mid well-being 21% Low well-being 11.3% Fathers: High well-being 89.9% Mid well-being 10.2% Low well-being 0% | CASP Score = B1 -Confidence intervals not reported |
| Stoneman *et al* (2007) | *N* = 50 mean age 37 yrs (s.d. 6.9) (married couples) Family income median $30 000 87% employed USA | *N* = 50 mean age 34 yrs (s.d. 5.7) Employed FT 38%, PT 26% | *N* = 50 (29 DS, 21 other ID) mean age 4.8 yrs 50% Male *N* = 32 mixed ID (unknown origin, fragile X, autism, Prader-willi syndrome, cerebral palsy) | Cross- sectional | ID: -ID Measure not reported -Temperament Assessment Battery Parental Mental Health: -Centre for Epidemiologic Studies Depression Scale -Questionnaire on Resources and Stress -Family Support Scale -Parental Attitudes towards Childrearing Questionnaire -Observed parenting | Recruited from early intervention programmes, pre-schools, parent groups and referrals from community members. | DS Mother depression (mean 7.43, s.d. 6.09, range 0–24) *v.* DS Father depression (mean 6.24, s.d. 5.34, range 0–21) Other ID Mother (mean 15.35, s.d. 10.13, range 3–38) *v.* Other Father (mean 10.47, s.d. 9.15, range 0–28). | CASP Score = B3 -ID measure and level of ID not reported -Comparison group contains some children with autism -95%CI not reported |
| McCarthy el al (2006) | *N* = 28 fathers Mean age 41.5 yrs (s.d. 6.91) Australia | *N* = 39 mothers Mean age 39.8 yrs (s.d. 5.3) | *N* = 40 children Mean age 10.4 yrs (s.d. 3.59) | Cross sectional | ID -Reported by family Parental Mental Health: -Brief Symptom Inventory (BSI) -Questionnaire on Resources and Stress | Recruited from Fragile X society | Mother *v.* Father BSI: M 52.77 (s.d. 9.93) *v.* M 54.93 (s.d. 9.14) QRS: M 19.87 (s.d. 9.11) *v.* M 18.18 (s.d. 8.55) | CASP Score = B2 -Level of ID not reported -95%CI not reported -Parents reported on child with highest support needs |
| Olsson *et al* (2006) | *N* = 179 fathers mean age 43 yrs (s.d. 6.3) (married couples) Sweden | *N* = 179 mothers ean age 39.8 yrs (s.d. 6.2) | *N* = 179 ID mean age 8.1 yrs (s.d. 4.3) 62% males | Cross-sectional | ID: -Parent reports Parental Mental Health: -Beck’s Depression Inventory -Division of 15 child-care tasks measure | Recruited from community-based programmes providing services to families of disabled children | Mothers wellbeing was more affected than fathers (F1,710 = 10.8, *P* < 0.05) when there was a child with ID in the family. Correlation between mothers’ and fathers’ BDI scores were *r* = 0.29, *P* < 0.05 in control families and *r* = 0.40, *P* < 0.05 in ID families. 15 | CASP Score = B2 -ID measured with parent reports -Level of ID not reported -Confidence intervals not reported |
| Hedov *et al* (2002) | *N* = 70 fathers Mean age 39.6 (s.d. 5.9) Sweden | *N* = 86 mothers Mean age 37.6 (s.d. 5.5) | *N* = Not reported Age 3.5–7 yrs (M 4.7) | Cross sectional | ID: -Not reported Parental Mental Health: -Parental Perception Inventory | Recruitment method not reported | 2 of 20 stress items were sig different between mothers and fathers: ‘Feeling worn out’ was higher in mothers, *P* < 0.05 ‘Concerned for spouses health’ was higher in fathers, *P* < 0.05 However, the same pattern was observed in control families | CASP Score = B3 -Recruitment method not reported -ID measure not reported -95%CI not reported |
| Olsson *et al* (2002) | *N* = 115 fathers Age not reported Sweden | *N* = 144 mothers Age not reported | *N* = 151 ID 0–16 yrs Mean age 7.4 yrs (s.d. 4.2) 60% males | Cross- sectional | ID: -parent reports Parental Mental Health: -Becks depression inventory | Recruited from community based programmes for families of disabled children | Mothers had higher depression scores than fathers: 9.2 (s.d. 7.4) *v.* 5.1 (s.d. 5.0) (t379 = 6.1, *P* < 0.01) | CASP Score = B2 -ID measured through parent reports -Confidence intervals not reported |
| Olsson *et al* (2001) | *N* = 265 120 ID fathers Age not reported Sweden | *N* = 145 ID mothers | *N* = 151 ID Mean age 7.4 yrs (s.d. 4.2) 60% males | Cross- sectional | ID: -Medical provider’s classification Parental Mental Health: -Becks Depression Inventory | Recruited from community-based programmes for families of children with disabilities | Fathers BDI scores were lower than mothers: 5 *v.* 9.2 Sig difference | CASP Score = B2 -Recruited from community programmes so may not represent families not accessing services -Parents age not reported -Confidence intervals not reported |
| Hedov *et al* (2000) | *N* = 165 *N* = 79 DS fathers Mean age 39.6 (s.d. 6.0) Sweden | *N* = 86 DS mothers Mean age 37.8 (s.d. 5.0) | *N* = DS Mean age 4.7 yrs | Cross- sectional | ID: -Not reported Parental Mental Health: -SF 36 mental health domain | Recruited through paediatrician | Father’s SF scores indicated better mental health than mothers: 79.2 (s.d. 17.9) *v.* 74 (s.d. 18.2) Sig difference | CASP Score = B1 -Confidence intervals not reported |

Table 4

| Author & Year | Father Characteristics | Son/daughter with/without ID | Study Design | Measures | Methodology | Results | Critique |
| --- | --- | --- | --- | --- | --- | --- | --- |
| Koza *et al* (2018) | *N* = 51 fathers Mean age 48.31 Poland | *N* = DS Mean age 13.25 48.41% Male | Cross sectional | ID: -Not reported Parental Mental Health: -Perceived Stress Scale -Oxford Happiness Questionnaire | Recruited from schools, rehabilitation centres and previous contacts | Significant predictors of wellbeing: Perceived stress (B = −0.38, *P* = 0.003) Ego-resiliency (B = 0.29, *P* = 0.012) Perceived social support and marital quality did not significantly predict wellbeing | CASP Score = B3 -ID measure not reported -ID level not reported -95%CI not reported |
| Norton *et al* (2016) | *N* = 62 fathers Mean age 39.06 (s.d. 8.53) USA | *N* = 62 DS Mean age 10.95 (s.d. 7.66) | Cross sectional | ID: -Not reported Parental Mental Health: -Hassles and Uplifts Scale Marital Quality: -Revised Dyadic Adjustment Scale -Revised Experiences in Close Relationships Questionnaire | Recruited through local and regional organisations and social media | Significant negative relationship between: Amount of respite and daily stress of fathers (B = −0.20, *P* < 0.05) No significant relationship: Marital quality and stress | CASP Score = B3 -ID measure not reported -ID level not reported -95%CI not reported |
| Giallo *et al* (2015) | *N* = 315 fathers Age not reported Australia | 315 children with ID plus one other condition (ASD, vision/hearing impairment, DS, cerebral palsy, epilepsy, psychiatric disorder, ADHD, brain injury, health problem) Mean age 7.8 yrs (s.d. 5.5) 69.8% male ID: 17.1% mild, 7.3% moderate, 2.2% severe, 0.3% profound, 73.1% not reported | Cross- sectional | ID: -measure not reported Parental Mental Health: -Depression Anxiety and Stress Scale -Developmental Behaviour Checklist -Parenting Hassles Scale -Parenting Sense of Competence Scale -Sources of Support Socioeconomic Factor: -Index of Relative Socio-economic status | Recruited from families involved in a previous study (Hudson *et al*, 2003; 2008). In the original study families recruited through schools and advertisement in newsletters of organizations specializing in support for families with children with ID | Mother and father in same family *N* = 220: -Depression Soioeconomic variables did not significantly predict fathers depression (R^2^ = 0.02, Adj R^2^ = 0.01, F4,310 = 1.39, *P* < 0.23). Factors sig predicting fathers depressive symptoms: child behaviour difficulties, parenting stress from child’s behaviours and needs, fathers own needs, low parenting satisfaction (R^2^ = 0.34, Adj R^2^ = 0.32, F11,303 = 14.24, *P* < 0.001). -Anxiety Socioeconomic variables did not predict fathers anxiety (R^2^ = 0.03, Adj R^2^ = 0.02, F4,314 = 2.21, *P* < 0.067) Factors sig predicting fathers anxiety symptoms: child behaviour difficulties, parenting stress arising from child’s behaviour difficulties, parenting stress arising from child’s behaviours and fathers’ own needs, low parenting satisfaction (R^2^ = 0.25, Adj R^2^ = 0.22, F11,303 = 9.05, *P* < 0.001) -Stress Socioeconomic variables did not predict fathers stress (R^2^ = 0.01, Adj R^2^ = 0.01, F4,314 = 0.74, *P* < 0.563) Factors sig predicting fathers stress: employment status, child behaviour difficulties, parenting stress arising from child’s behaviour difficulties, parenting stress arising from child’s needs and fathers’ own needs, low parenting satisfaction (R^2^ = 0.39, Adj R^2^ = 0.36, F11,303 = 17.21, *P* < 0.001) | CASP Score = B3 -Father sample from those enrolled to participate in a child behaviour management programme- so not representative -Includes children with ID + ASD in the sample -Mental health score based on distress in the past week |
| Norlin *et al* (2013) | *N* = 46 fathers Mean age 35.3 (s.d. 6.1) Sweden | *N* = 58 Mean age 41 months (s.d. 27.8) 62.1% males | Cross- sectional | ID: -Not reported Parental Mental Health: -BDI-2r | Recruited by staff at service centres for families of children with disabilities | Mental health was significantly predicted by -Marital quality (t = −2.84, *P* < 0.01) -High economic risk (t = 2.16, *P* < 0.05) -Problem behaviours (t = 3.06, *P* < 0.01) | CASP Score = B2 -Did not report ID measure used -Confidence intervals not reported |
| Hartley *et al* (2012) | *N* = 105 fathers (59 fathers of child with DS, 46 fathers of child with FXS) Age not reported USA | DS *N* = 59 mean age 15.2 yrs (s.d. 3.0) 69.6% male FXS *N* = 46 mean age 15.6 yrs (s.d. 2.6) 79.5% male ASD *N* = 135 mean age 16.0 yrs (s.d. 2.8) 72.3% male | Cross- sectional | ID: -Medical reports & genetic testing -Wide Range Intelligence Test -Vineland screener -Stanford-Binet Intelligence Scale -Autism behaviour checklist -Scales of independent behaviour revised Parental Mental Health: -Centre of Epidemiological Studies Depression Scale -Questionnaire of resources and stress -Multidimensional coping inventory | Recruited from local media advertisements, newsletters to disability organisations, brochures and postings in clinics. Disability listser*v.* and a university research registry. | -No sig difference in use of emotion-focused or problem-focused coping by diagnostic group. -Regression model: Additional children with a disability and higher level of maternal depressive symptoms were sig positive predictors of paternal depression. Model predicted 15% of variance in depression and 20% variance in pessimism. | CASP Score = B3 -95%CI not reported -Some of ASD group had ID -Behaviour problems reported by mothers rather than fathers. |
| Griffiths *et al* (2011) | *N* = 39 Fathers: -Angelman syndrome = 12 -Cornelia de Lange syndrome = 14 -Cri du chat syndrome = 13 | Angelman syndrome *N* = 15 Mean age 10.07 (4.79) Cornelia de Lange syndrome *N* = 16 Mean age 11.75 (s.d. 3.49) Cri du chat syndrome *N* = 18 Mean age 7.83 (s.d. 4.66) | Cross- sectional | ID: -Vineland adaptive behaviour scale (VABS-II) Parental Mental Health: -Questionnaire on Resources and Stress-Short form | Recruited from a database held by research team, and national parent support groups | Anxiety: -Angelman syndrome M 10.42 (s.d.4.72) -Cornelia de Lange M 5.85 (s.d. 4.35) -Cri du Chat M 9.0 (s.d. 4.16)  Depression -Angleman syndrome M 8.50 (s.d. 4.49) -Cornelia de Lange M 4.29 (s.d. 2.62) -Cri du Chat M 6.92 (s.d. 4.09)  Stress -Angleman syndrome M 20.75 (s.d. 10.07) -Cornelia de Lange M 16.18 (s.d. 98.67) -Cri du Chat M 16.68 (s.d. 5.52) | CASP Score = B3 -Level of ID not reported -Confidence intervals not reported -Small number of fathers in sample |
| Rowbotham *et al* (2011) | *N* = 12 fathers 45–55 yrs (with the exception of one father aged 55–65 yrs) Australia | *N* = 12 children Mean age 24 yrs (range: 18–30 yrs) 58% females Problem behaviour exhibited by the children did not differ between mothers and fathers, and was within the average range. | Cross- sectional | ID: -Adaptive Behavioural Scale (as reported by parents) Parental Mental Health: -Carers’ Assessment of Difficulties Index (CADI) - Carers’ Assessment of Satisfactions Index (CASI) - Hassles and Uplifts Scale - General Health Questionnaire-28 (GHQ-28) Social Support Factor: -‘How satisfied with partners caregiving and emotional support?’ Likert scale | Recruited through public and private-sector groups providing services to carers of adults with intellectual disabilities | For fathers, none of the measures were significantly related to care-giving satisfaction. -Hassles and Uplifts For fathers, there was a significant positive correlation between Work and Partner Hassles (q = 0.68, *P* = 0.008, one-tailed). The Uplift sub-scales of Work and Home Duties were positively correlated for mothers (q = 0.55, *P* = 0.03, one-tailed), while there were significant, positive inter- correlations for all three sub-scales for fathers (Partner and Work, q = 0.86, *P* < 0.001; Partner and Home Duties, q = 0.54, *P* = 0.037; Work and Home Duties, q = 0.65, *P* = 0.011, all one-tailed). | CASP Score = B4 -Small sample of parents known to services -Level of ID not reported |
| Dabrowska *et al* (2010) | DS *N* = 27 fathers Mean age 34.9 (s.d. 6.1) | Autism *N* = 26 −65.4% males Mean age 4.8 (s.d. 1.35) −34.6% females Mean age 4.7 (s.d. 1.34) DS *N* = 27 −55.6% males Mean age 4.3 (s.d. 1.58) −44.4% females Mean age 4.3 (s.d. 1.48) TD *N* = 29 −34.5% males Mean age 4.2 (s.d. 1.23) −65.5% females Mean age 4.9 (s.d. 1.42) | Cross- sectional | Parental Mental Health: -Questionnaire of Resources and Stress (QRS) - Coping Inventory for Stressful Situations (CISS) Socioeconomic Factor: -Questionnaire of Resources and Stress for Families with Chronically Ill or Handicapped Members short form | Recruited from centres for early intervention (autism or DS), therapy centres (autism) or kindergar- tens (DS and TD). | No significant difference in coping styles between groups | CASP Score = B3 -95%CI not reported -Small sample size -Level of ID not reported -Young children in sample so possible not all those with ID have been identified. |
| Emmerson *et al* (2010) | Wave one: *N* = 18 552 families Wave two: *N* = 15 590 families Wave three: *N* = 15 246 families Age not reported UK | Wave one = 9 mths old Wave two = 3 yrs old Wave three = 5 yrs old | Longitudinal | ID: -Bracken Basic Concept Scale -Naming Vocabulary subscale from British Ability Scale II Parental Mental Health: -K6 Scale Socioeconomic Factor: -Highest qualification, employment status, social class, income poverty, housing situation, welfare benefits, material hardship | Secondary data analysis of Millennium Cohort Study (waves 1–3) Families eligible to receive child benefit, born 09/2000 and 01/2002 | Matching on the basis of socioeconomic circumstances: -Fathers: reduced between group differences by 45% to 11%. Severe Delay: Fathers more likely to be at risk of psychiatric disorder than fathers of no delay only when child was 5 years old (14% *v.* 8%, OR = 1.82, 95%CI 1.05–3.15, *P* < 0.031) Less severe delay: Fathers more likely to be at risk of psychiatric disorder than fathers of no delay only when child was 3 (14% VS 7%, OR = 1.93, 95% CI = 1.10–3.42) and 5 years old (15% *v.* 8%, OR = 1.99, 95%CI 1.10–3.61, *P* = 0.020). | CASP Score = B3 - Young children in sample so possible not all those with ID have been identified -Follow-up not long enough to identify ID diagnosed later in development -Level of ID not reported |
| Foster *et al* (2010) | *N* = 15 fathers Mean age 42.07 yrs (s.d. 9.85) USA | *N* = Not reported Smith-Magenis Syndrome | Cross sectional | ID: Parental Mental Health: -Center for epidemiologic studies depression scale -Becks Anxiety Inventory -Caregiver well-being scale Parental Perceptions: -Psychosocial impact scale (benefit finding subscale) | Recruited through parent and researcher Smith-Magenis syndrome list serve | Fathers who perceived more benefits of having a child with SMS reported higher carer well-being (*r* = 0.67, *P* < 0.01). | CASP Score = C1,2 -Small number of fathers in sample -Confidence intervals not reported |
| MacDonald *et al* (2010) | *N* = 99 fathers Age not reported Ireland | *N* = 83 children (53 DS, 30 other ID) Age not reported | Cross sectional | ID: *-Not reported* Parental Mental Health -Parent and Family Problems subscale of Questionnaire on Resources and Stress -Hospital Anxiety and Depression scale | Recruited from ID services | Fathers of children with DS reported lower stress levels: (M 3.02, s.d. 3.43) than fathers of children with other types of ID: (M 5.00, s.d. 4.63) t(82) = 2.44, *P* = 0.019 Fathers whose partner worked outside the home reported lower anxiety (M 11.10, s.d. 4.10) than fathers whose partner didn’t work (M 13.32, s.d. 4.30) T(97) = 2.39, *P* = 0.019 | CASP Score = B1 -95% CI’s not reported -Level of ID not reported -parent age not reported |
| Gerstein *et al* (2009) | *N* = 115 fathers Age not reported (married couples) USA | *N* = 115 ID 3 yrs old | Longitudinal (child aged 36–60 months) | ID: -Bayley Scales of Infant Development II Parent Mental Health: -Parenting Daily Hassles measure -Symptom Checklist-35 -Dyadic Adjustment Scale -Parent Child Interaction Rating Scale (tested at 36, 48, 60 months) | Recruited from community agencies serving families of children with ID | Marital adjustment was a protective factor against poor father mental health at 36 months (B = −0.235 (SE = 0.058), *P* = <0.001) | CASP Score = B2 -Level of ID not reported -Parental age not reported -95%CI not reported |
| Olsson *et al* (2008) | *N* = 49 fathers Mean age 36.2 yrs (s.d. 6.4) Sweden | *N* = 62 0–5 yrs | Cross- sectional | ID: -Reported by centres Parental Mental Health: -Beck’s Depression Inventor-2r -Socioeconomic hardship: List of items parent would like but could not afford (adapted from Emerson *et al*, 2006) | Recruited from centres providing support to families of children with disabilities | The greater economic hardship, the higher the risk of poor wellbeing Model predicted 42% of the variance for fathers | CASP Score = B1 -Confidence intervals not reported |
| Stoneman *et al* (2007) | *N* = 50 mean age 37 yrs (s.d. 6.9) (married couples) Family income median $30 000 87% employed USA | *N* = 50 (29 DS, 21 other ID) mean age 4.8 yrs 50% Male *N* = 32 mixed ID (unknown origin, fragile X, autism, Prader-willi syndrome, cerebral palsy) | Cross- sectional | ID: -Temperament Assessment Battery Parental Mental Health: -Centre for Epidemiologic Studies Depression Scale -Questionnaire on Resources and Stress -Family Support Scale -Parental Attitudes towards Childrearing Questionnaire -Observed parenting Socioeconomic Factor: -Family income | Recruited from early intervention programmes, pre-schools, parent groups and referrals from community members. | The ‘DS advantage’ disappeared after income differences between groups were controlled. Father’s reports of depression were predicted by lower family income (R^2^ = 0.11, Adj R^2^ = 0.08, F?? = 2.17, *P* < 0.05) | CASP Score = B3 -ID measure and level of ID not reported -Comparison group contains some children with autism -95%CI not reported |
| McCarthy el al (2006) | *N* = 28 fathers Mean age 41.5 yrs (s.d. 6.91) Australia | *N* = 28 children Mean age 10.4 yrs (s.d. 3.59) | Cross sectional | ID: -Reported by family Parental Mental Health: -Brief Symptom Inventory (BSI) -Questionnaire on Resources and Stress | from Fragile X society | Child behaviour was the best predictor of BSI, accounting for 50% of the regression variance F(3,21) = 8.95, *P* < 0.001 | CASP Score = B2 -Level of ID not reported -95%CI not reported -Parents reported on child with highest support needs |
| Olsson *et al* (2006) | *N* = 375 fathers 179 ID fathers mean age 43 yrs (s.d. 6.3) 196 TD fathers mean age 42yrs  (s.d. 6.9) (married couples) Sweden | *N* = 179 ID mean age 8.1 yrs (s.d. 4.3) 62% males *N* = 196 TD mean age 8.2 yrs (s.d. 4.4) 62% males | Cross-sectional | ID: -Parent reports Parental Mental Health: -Beck’s Depression Inventory -Division of 15 child-care tasks measure Socioeconomic Factor: -Level of involvement in paid work | Recruited from community-based programmes providing services to families of disabled children | For fathers, well-being increased with higher involvement in paid work | CASP Score = B2 -ID measured with parent reports -Level of ID not reported |
| Olsson *et al* (2002) | *N* = 115 fathers Age not reported Sweden | *N* = 151 ID 0–16 yrs Mean age 7.4 yrs (s.d. 4.2) 60% males | Cross- sectional | ID: -parent reports Parental Mental Health: -Becks depression inventory Parental Perceptions: -Swedish version of the short Sense of Coherence scale | Recruited from community based programmes for families of disabled children | Sense of coherence did not predict poor mental health (correlation *r* = −0.65) | CASP Score = B2 -ID measured through parent reports -Confidence intervals not reported |
